# Barriers, adoption patterns, and determinants of artificial intelligence use among health sciences students in Palestine

**DOI:** 10.1371/journal.pdig.0001575

**Published:** 2026-08-06

**Authors:** Nesreen Alqaissi, Mohammad Qtait

**Affiliations:** Nursing College, Palestine Polytechnic University, Hebron, Palestine; University of Toronto - St George Campus: University of Toronto, CANADA

## Abstract

Artificial intelligence (AI) is increasingly integrated into healthcare education worldwide, yet disparities in access, training, and institutional readiness remain evident, particularly in low-resource and conflict-affected settings. Understanding how health sciences students engage with AI technologies and the barriers they encounter is essential for guiding the development of AI-ready curricula in Palestinian universities. This study aimed to examine the adoption patterns, perceived barriers, and determinants of artificial intelligence use among health sciences students at Palestine Polytechnic University in Palestine. A descriptive cross-sectional study was conducted among 666 undergraduate students from the Colleges of Nursing, Medicine and Health Sciences, and Dentistry. Data were collected using a validated self-administered questionnaire assessing demographic characteristics, AI knowledge, attitudes, practice behaviors, and perceived barriers. Descriptive statistics summarized usage patterns. Mann–Whitney U tests, Kruskal–Wallis tests, and chi-square analyses examined group differences. Multivariate logistic regression identified predictors of AI adoption. Statistical significance was set at p ≤ .05. The result of the study. AI use was highly prevalent, with 93.4% of students reporting active engagement. AI was primarily used for study and learning (87.7%), written assignments (57.5%), and personal purposes (54.2%). Significant differences in AI usage were observed across academic disciplines (χ² = 17.292, p = .008), with dentistry students reporting longer daily use. Major barriers included limited curriculum integration (48.2%), ethical and privacy concerns (47.9%), and insufficient training centers (40.4%). Multivariate analysis showed that college affiliation and knowledge score significantly predicted AI adoption, whereas gender, academic year, and previous AI training were not significant predictors. The Conclusion. Despite widespread exposure to AI technologies, students’ engagement remains largely informal and constrained by curricular, infrastructural, and ethical barriers. Institutional strategies including curriculum reform, faculty development, and improved digital infrastructure are necessary to support responsible AI integration in health sciences education in Palestine.

## Introduction

Artificial intelligence (AI) has emerged as a transformative technology in contemporary healthcare, influencing diagnostic processes, treatment planning, health system management, and professional education. Advances in machine learning, natural language processing, and generative AI have enabled automated clinical decision support, predictive analytics, and personalized patient care [1]. As these technologies continue to expand, healthcare professionals are increasingly expected to develop competencies in interpreting AI outputs, evaluating algorithmic recommendations, and applying digital tools responsibly within clinical practice. Consequently, health professions education worldwide is beginning to integrate AI-related competencies into undergraduate curricula through simulation platforms, adaptive learning systems, and AI-assisted tutoring tools [2,3].

Despite the growing global momentum toward AI integration in healthcare education, substantial disparities persist in students’ preparedness, confidence, and practical use of AI technologies. Studies conducted among medical and health sciences students indicate that while learners generally demonstrate positive attitudes toward AI and recognize its potential value in healthcare, their knowledge and structured training remain limited [4,5]. Many students report relying on informal sources such as peer networks, online tutorials, or social media to learn about AI due to the absence of formal curricular integration [6]. This gap between interest and structured training raises concerns regarding the safe and responsible application of AI in clinical education and professional practice.

Emerging evidence also indicates that high levels of AI use among students do not necessarily correspond with adequate understanding of its capabilities and limitations. Recent studies suggest that generative AI tools are frequently used for academic writing, summarization, and information retrieval rather than for deeper clinical reasoning or evidence synthesis [7]. Scholars have warned that unsupervised reliance on AI systems may increase the risk of misinformation, algorithmic bias, and diminished clinical reasoning if students lack the critical skills necessary to evaluate AI-generated outputs [8]. Therefore, integrating AI literacy into health sciences education is increasingly considered essential for ensuring responsible and effective use of digital health technologies.

Within low-resource and conflict-affected contexts, the integration of AI into health professions education faces additional structural challenges. Limited technological infrastructure, inconsistent internet access, and financial barriers to advanced digital tools may restrict students’ exposure to AI platforms and training opportunities. In such settings, institutional readiness—including faculty expertise, curriculum design, and digital infrastructure—plays a critical role in shaping adoption patterns [9]. These contextual constraints may contribute to disparities in AI literacy and limit students’ ability to engage meaningfully with emerging digital health technologies.

The Palestinian higher education system represents a particularly relevant context for examining these issues. Universities in Palestine operate within a complex environment characterized by limited resources, infrastructural constraints, and periodic disruptions related to political instability. Although digital technologies have increasingly been introduced into Palestinian universities, the integration of AI within health sciences education remains largely informal and fragmented. Preliminary studies conducted among Palestinian university students indicate widespread awareness and use of generative AI tools such as ChatGPT; however, students frequently report limited formal training and uncertainty regarding ethical use, data privacy, and academic integrity [10,11].

In addition to infrastructural challenges, ethical concerns surrounding AI technologies have become a major issue in health professions education. These concerns include risks related to patient data privacy, algorithmic bias, transparency of AI decision-making processes, and potential overreliance on automated systems [12]. Addressing these issues requires integrating digital ethics and responsible AI use into health sciences curricula to ensure that future healthcare professionals are prepared to critically evaluate and safely implement AI technologies in clinical practice [13].

Although a growing number of studies have examined knowledge, attitudes, and perceptions toward AI among health sciences students, research exploring the combined influence of adoption patterns, barriers, and determinants remains limited. In particular, there is a lack of empirical evidence examining how demographic characteristics, institutional context, training exposure, and perceived barriers interact to shape AI adoption behaviors in low-resource educational settings. Understanding these factors is essential for developing effective strategies to integrate AI competencies into health professions education.

Therefore, this study aimed to examine the barriers, adoption patterns, and determinants of AI use among health sciences students at Palestine Polytechnic University. By analyzing students’ usage behaviors, perceived obstacles, and demographic determinants, the study seeks to provide evidence that can guide curriculum development, faculty training initiatives, and institutional strategies for integrating AI into undergraduate health education in Palestine.

### Literature review

The integration of artificial intelligence (AI) into health professions education has expanded rapidly over the past decade as digital technologies increasingly shape biomedical research, clinical decision-making, and healthcare delivery. AI systems—including machine learning algorithms, natural language processing, and generative models—are now widely applied in clinical diagnostics, medical imaging, precision medicine, and healthcare management [1]. These technological advancements have prompted growing recognition that future healthcare professionals must acquire competencies in AI literacy, including understanding algorithmic outputs, critically evaluating AI-generated information, and integrating digital tools into evidence-based clinical practice [14]. Consequently, universities worldwide are gradually introducing AI-related learning experiences within medical, nursing, and allied health curricula.

Several studies have explored students’ knowledge, attitudes, and adoption behaviors regarding AI in health sciences education. Research conducted among medical and health sciences students in Jordan found that although most students were aware of AI technologies and expressed positive attitudes toward their potential clinical applications, only a minority had received formal training or structured instruction in AI [4]. Similar findings have been reported in Saudi Arabia, where health sciences students demonstrated high interest in AI but limited practical knowledge of its technical foundations and ethical implications [5]. These findings suggest that enthusiasm for AI adoption often exceeds students’ actual preparedness to use AI tools effectively.

In addition to knowledge gaps, studies consistently report that students tend to use AI primarily for basic academic tasks rather than advanced clinical applications. For example, research among Egyptian medical students found that generative AI tools were most commonly used for text summarization, grammar correction, and assignment preparation rather than clinical reasoning or diagnostic support [7]. Similarly, multinational surveys across the Middle East indicate that students frequently rely on AI systems for information retrieval and academic writing support but rarely engage with AI-driven clinical simulation or diagnostic applications [6]. This pattern suggests that AI adoption in health sciences education often remains superficial, reflecting opportunistic use rather than structured pedagogical integration.

Recent scholarship has also emphasized that high levels of AI use do not necessarily indicate adequate understanding of its underlying mechanisms or limitations. Generative AI tools, particularly large language models, can produce convincing but occasionally inaccurate outputs, raising concerns regarding misinformation, hallucination errors, and overreliance on automated systems in clinical learning environments [8]. Without appropriate educational guidance, students may adopt AI tools without fully understanding their potential biases or limitations, which could negatively influence clinical reasoning and evidence-based practice.

Beyond educational applications, AI is rapidly transforming biomedical research and clinical practice. In medical diagnostics, AI-driven algorithms have demonstrated significant potential in areas such as cardiovascular imaging, radiology, and pathology, where machine learning models can assist clinicians in identifying complex patterns within large datasets [2]. In biomedical research, computational approaches such as bioinformatics and network pharmacology are increasingly used to explore disease mechanisms, identify therapeutic targets, and support drug discovery processes. These developments illustrate the expanding role of AI technologies across the biomedical sciences and reinforce the importance of preparing health sciences students to engage with complex data-driven systems.

Multiple determinants influence students’ adoption of AI technologies in health professions education. Individual factors such as digital literacy, technological self-efficacy, and perceived usefulness of AI tools have been shown to influence students’ willingness to adopt AI in learning and research activities [15,16]. Institutional factors—including curriculum integration, faculty expertise, and access to technological infrastructure—also play a significant role in shaping students’ exposure to AI. Universities that incorporate AI modules, simulation laboratories, or digital health courses report higher levels of student engagement and more advanced forms of AI application in academic tasks [9].

Barriers to AI integration remain a central theme in the literature. Common obstacles identified across international studies include lack of formal training, limited access to AI platforms, insufficient institutional support, and uncertainty regarding ethical and legal considerations [17,18]. Ethical concerns are particularly prominent among health sciences students, who often express apprehension regarding data privacy, algorithmic bias, and the potential erosion of professional judgment in clinical decision-making [12]. Addressing these concerns requires integrating ethical guidelines and responsible AI use into health professions education to ensure safe and accountable application of digital technologies.

These challenges may be more pronounced in low-resource and conflict-affected settings, where universities often face additional constraints related to infrastructure, technological access, and faculty training capacity. In such contexts, the digital divide can limit students’ exposure to advanced AI tools and restrict opportunities for practical engagement with emerging technologies. Preliminary studies among Palestinian university students indicate growing awareness and use of AI tools, particularly generative AI systems, but limited institutional support and lack of formal training remain major barriers to effective adoption [10, 11].

Overall, the existing literature demonstrates that AI adoption among health sciences students is shaped by a complex interaction of individual, institutional, and contextual factors. While global interest in AI education continues to increase, significant gaps remain in understanding how barriers, adoption patterns, and demographic determinants interact within specific educational environments. In the Palestinian context, empirical research examining these relationships remains scarce. Addressing this gap is essential for informing evidence-based strategies to integrate AI competencies into health sciences education and to support the development of a digitally competent healthcare workforce.

## Methods

### Study design

This study employed a descriptive cross-sectional design to examine barriers, adoption patterns, and determinants of artificial intelligence (AI) use among undergraduate health sciences students at Palestine Polytechnic University (PPU). Cross-sectional designs are commonly used in health education research to assess knowledge, behaviors, and perceptions within a defined population at a single time point and to identify factors associated with technology adoption [4,3].

### Study setting and population

The study was conducted at Palestine Polytechnic University in Hebron, Palestine, which hosts several academic programs preparing students for professional roles in healthcare. Data were collected from students enrolled in three colleges: the College of Nursing, the College of Medicine and Health Sciences, and the College of Dentistry.

The target population consisted of all undergraduate students registered in these colleges during the 2024–2025 academic year. Students were eligible to participate if they were currently enrolled in one of the health sciences programs and consented to participate voluntarily. Students who were on academic leave during the data collection period or who submitted incomplete questionnaires were excluded from the analysis.

### Sample size and sampling strategy

A total of 666 students participated in the study. The sample included students from nursing (36.3%), medicine and health sciences (36.8%), and dentistry (26.9%) programs. A convenience sampling approach was used because the survey was distributed electronically to all eligible students through the university learning platform during the data collection period. This approach allowed broad participation across academic levels and disciplines within the institution.

### Instrument development

Data were collected using a structured self-administered questionnaire designed to assess students’ knowledge, attitudes, practices, and perceived barriers regarding the use of artificial intelligence in health sciences education. The questionnaire was developed based on previously published instruments and recent studies examining AI adoption in health professions education [4,9,3].

The instrument consisted of five domains:

1. **Demographic and usage characteristics**

This section collected background information including gender, age, academic year, college affiliation, and previous exposure to AI training. It also included questions about frequency of AI use, daily duration of use, and purposes of AI use.

2. **AI Knowledge**

Knowledge was measured using seven dichotomous items (true/false) assessing students’ understanding of basic AI concepts, applications, and limitations. Higher scores indicated greater foundational AI literacy.

3. **Attitudes toward AI**

Attitudes were measured using ten Likert-scale items ranging from 1 (strongly disagree) to 5 (strongly agree), assessing perceived usefulness, safety, and future relevance of AI in healthcare.

4. **AI practice and adoption behaviors**

Practice was measured using seven items rated on a five-point frequency scale (1 = never to 5 = always). These items assessed how frequently students used AI for activities such as studying, assignment preparation, information retrieval, and clinical learning.

5. **Barriers to AI adoption**

This section included seven items evaluating structural, educational, and ethical barriers such as lack of training opportunities, limited curriculum integration, concerns regarding privacy and ethics, technological complexity, and lack of institutional resources.

### Validity and reliability

Content validity was assessed through expert evaluation. The questionnaire was reviewed by specialists in nursing education, digital health, and medical informatics to ensure clarity, relevance, and cultural appropriateness. The experts evaluated each item for relevance and clarity, and modifications were made based on their recommendations.

Construct validity was evaluated using item–total correlation analysis, demonstrating statistically significant associations between items and their respective domains (p < .05). In addition, exploratory factor analysis was performed to assess the underlying structure of the questionnaire domains and confirm the grouping of items into the intended constructs.

Internal consistency reliability was assessed using Cronbach’s alpha coefficient. The overall Cronbach’s alpha for the instrument was 0.763, indicating acceptable internal consistency for research purposes.

### Data collection procedure

Data collection was conducted during the Fall 2025 semester over a four-week period. The survey was distributed electronically through the university’s official learning management system to ensure accessibility to all eligible students. Participation was voluntary, and students were informed about the purpose of the study before providing electronic consent. The questionnaire was completed anonymously, and no personally identifiable information was collected.

### Ethical considerations

Ethical approval for this study was obtained from the Institutional Review Board (IRB) of Palestine Polytechnic University (Approval No. EA-023-2025). Participation was voluntary, and informed electronic consent was obtained from all participants prior to completing the questionnaire. Students were informed about the purpose of the study, confidentiality of responses, and their right to withdraw at any time without penalty. No personally identifiable information was collected, and all data were stored securely and analyzed anonymously in accordance with established ethical standards for research involving human participants.

### Data analysis

Data were analyzed using SPSS version 20. Descriptive statistics, including frequencies, percentages, means, and standard deviations, were used to summarize demographic characteristics, patterns of AI use, and perceived barriers.

The Kolmogorov–Smirnov test was applied to assess the normality of distribution for continuous variables. As the data were not normally distributed, non-parametric tests were used to examine group differences. Mann–Whitney U tests were applied to compare two-group variables such as gender and prior AI training, while Kruskal–Wallis tests were used to examine differences across multiple groups including academic year and college affiliation. Chi-square analysis was conducted to assess associations between college affiliation and frequency of AI use.

To identify independent predictors of AI adoption, a multivariate logistic regression analysis was performed including gender, academic year, college affiliation, prior AI training, and knowledge score as explanatory variables. Adjusted odds ratios (ORs) and 95% confidence intervals were calculated to determine the strength of associations. Statistical significance was set at p ≤ .05.

## Results

### Participant characteristics

A total of 666 undergraduate health sciences students participated in the study. The demographic characteristics of the participants are presented in Table 1. The sample was predominantly female (73.9%), while males represented 26.1% of the participants. The mean age of the respondents was 20 ± 1.83 years, reflecting the typical age range of undergraduate students in health sciences programs.

**Table 1:** Demographic characteristics of the sample (n = 666).

Variable	Category	n	%
Gender	Female	492	73.9
	Male	174	26.1
Age	Mean ± SD	20 ± 1.825	—
College	Nursing	242	36.3
	Medicine & Health Sciences	245	36.8
	Dentistry	179	26.9
Academic Year	First	162	24.3
	Second	174	26.1
	Third	153	23.0
	Fourth	131	19.7
	Fifth	44	6.6
	Sixth	2	0.3
Use of AI Tools	Yes	622	93.4
	No	44	6.6

Participants were distributed across three academic colleges: Nursing (36.3%), Medicine and Health Sciences (36.8%), and Dentistry (26.9%). Regarding academic level, students from the first (24.3%) and second years (26.1%) constituted the largest proportions of the sample.

Importantly, AI tool usage was highly prevalent, with 93.4% of students reporting active use of AI technologies, while only 6.6% reported not using AI tools. This finding indicates widespread exposure to AI applications among health sciences students (Table 1).

### Patterns of AI usage

Students reported using AI tools for a variety of academic and personal purposes. As shown in Table 2, the most common application was study and learning support, reported by 87.7% of respondents. This indicates that AI tools are widely used to assist students in understanding course material and enhancing their learning process.

**Table 2:** AI usage purposes among health sciences students (n = 622).

Purpose of AI Use	n	%
Study & learning	537	87.7
Research	295	48.2
Professional tasks	130	21.2
Personal use	332	54.2
Written assignments	352	57.5
Other	24	3.9

More than half of the participants (57.5%) reported using AI tools for written assignments, suggesting that generative AI technologies are commonly used for academic writing assistance. Additionally, 54.2% of students reported personal use, while 48.2% used AI for research-related tasks, such as literature exploration or topic clarification.

In contrast, professional or discipline-specific use of AI was reported by only 21.2% of students, indicating that AI tools are still rarely used for clinical or professional learning activities (Table 2).

### Daily duration of AI use

The amount of time students spent using AI tools for educational and research purposes is presented in Table 3. Most students reported relatively short daily engagement with AI technologies.

**Table 3:** Daily time spent using AI for educational and research tasks.

Time Category	n	%
< 30 minutes	208	34.0
30 minutes – 1 hour	209	34.2
1–2 hours	116	19.0
> 2 hours	78	12.8

Approximately 34.0% of students used AI for less than 30 minutes per day, while 34.2% reported using AI for 30 minutes to one hour daily. A smaller proportion (19.0%) used AI tools for one to two hours per day, whereas only 12.8% reported using AI for more than two hours daily.

These findings suggest that AI usage among students is frequent but typically limited to short, task-oriented interactions rather than extended usage (Table 3).

### Differences in AI usage across colleges

AI usage patterns differed across academic disciplines. As illustrated in Table 4, students from the College of Nursing and the College of Medicine and Health Sciences most frequently reported AI use for less than one hour daily.

**Table 4:** Frequency of using AI tools by college.

College	<30 min	30–60 min	1–2 hours	>2 hours	Total
Nursing	67 (32.2%)	72 (34.4%)	30 (25.9%)	25 (32.1%)	194
Medicine & Health Sciences	85 (40.9%)	91 (43.5%)	44 (37.9%)	20 (25.6%)	240
Dentistry	56 (26.9%)	46 (22.0%)	42 (36.2%)	33 (42.3%)	177

In contrast, dentistry students demonstrated higher levels of extended AI engagement, with 42.3% reporting usage exceeding two hours per day. This finding suggests that disciplinary context may influence students’ engagement with AI technologies.

To determine whether these differences were statistically significant, a Chi-square test was conducted. The results demonstrated a significant association between college affiliation and AI usage frequency (χ² = 17.292, df = 6, p = .008), indicating that students’ daily engagement with AI tools varies significantly across academic disciplines (Table 5).

**Table 5:** Chi-square test for differences in AI usage by college.

Test	Value	df	p-value
Pearson Chi-square	17.292	6	.008

### Barriers to AI adoption

Students reported several barriers that limit the effective integration of AI technologies in health sciences education. As shown in Table 6, the most frequently reported barrier was limited curriculum integration, identified by 48.2% of participants.

**Table 6:** Barriers to AI adoption in health sciences education.

Barrier	n	%
Lack of knowledge/expertise	257	38.6
Lack of access/equipment	199	29.9
Ethical & privacy concerns	319	47.9
Lack of time	232	34.8
Complexity of AI	188	28.2
Limited curriculum integration	321	48.2
Lack of teaching centers	269	40.4

Similarly, ethical and privacy concerns were reported by 47.9% of students, highlighting concerns related to data protection, algorithmic bias, and responsible use of AI systems.

Other important barriers included lack of teaching centers (40.4%), lack of knowledge or expertise (38.6%), and time constraints (34.8%). Additionally, 29.9% of students reported limited access to equipment, and 28.2% indicated that AI systems were too complex to use effectively.

These findings demonstrate that both educational and structural factors hinder meaningful AI adoption in health sciences education (Table 6).

### Determinants of AI adoption

Non-parametric tests were used to examine differences in AI adoption scores across demographic groups. As presented in Table 7, college affiliation was the only variable significantly associated with AI practice scores (H = 18.100, p < .001). Dentistry and medicine students reported higher AI practice scores compared with nursing students.

**Table 7:** Determinants of AI adoption based on practice scores.

Variable	Group	Mean Score	Test Statistic	p-value
Gender	Female	3.58	U = –1.617	.106
	Male	3.57		
Academic Year	Various	—	H = 4.520	.476
College	Nursing	3.18	H = 18.100	<.001
	Medicine	3.38		
	Dentistry	3.44		
AI Training	Yes	3.30	U = –0.244	.807
	No	3.23		

In contrast, gender (p = .106), academic year (p = .476), and prior AI training (p = .807) showed no statistically significant differences in AI adoption scores, Table 7.

### Multivariate predictors of AI adoption

A multivariate logistic regression analysis was performed to identify independent predictors of AI adoption while controlling for potential confounding variables. The results are presented in Table 8.

**Table 8:** Multivariate logistic regression predicting AI adoption.

Variable	Adjusted OR	95% CI	p-value
Gender (Male vs Female)	1.12	0.82–1.53	.41
Academic year	1.04	0.92–1.17	.52
Medicine vs Nursing	1.37	1.02–1.84	.034
Dentistry vs Nursing	1.69	1.21–2.36	.002
Previous AI training	1.18	0.86–1.63	.30
Knowledge score	1.26	1.10–1.45	.001

The analysis showed that college affiliation and knowledge score were significant predictors of AI adoption. Students from dentistry (OR = 1.69, p = .002) and medicine (OR = 1.37, p = .034) had significantly higher odds of AI adoption compared with nursing students.

Additionally, knowledge score was positively associated with AI adoption (OR = 1.26, p = .001). However, gender, academic year, and previous AI training were not statistically significant predictors.

## Discussion

This study examined barriers, adoption patterns, and determinants of artificial intelligence (AI) use among health sciences students at Palestine Polytechnic University. The findings reveal widespread engagement with AI tools, yet they also highlight significant structural, educational, and ethical challenges that limit meaningful integration of AI into health sciences education in Palestine. These results contribute to the growing body of literature examining AI adoption in medical education, particularly within resource-constrained and conflict-affected contexts.

A key finding of the present study is the extremely high prevalence of AI use among students, with 93.4% reporting active engagement with AI tools. This level of adoption is comparable to recent international studies indicating that generative AI systems, particularly large language models, have rapidly become part of students’ daily academic practices [2,3]. However, the purposes of AI use identified in this study suggest that engagement remains largely limited to academic tasks such as studying, assignment preparation, and personal information retrieval. Similar usage patterns have been documented among health sciences students in Jordan and Egypt, where AI tools are commonly used for text summarization, writing assistance, and general learning support rather than clinical reasoning or advanced data analysis [4,7]. This pattern indicates that AI adoption among students may reflect convenience-driven usage rather than structured educational integration.

Importantly, the results reveal a notable paradox between widespread AI use and limited knowledge or expertise. Although most students reported using AI regularly, more than one-third identified lack of knowledge or expertise as a major barrier. This discrepancy suggests the presence of an “adoption-without-understanding” phenomenon, in which students rely on AI tools despite limited comprehension of their underlying mechanisms or limitations. Similar concerns have been raised in recent medical education research, which warns that unsupervised reliance on generative AI systems may increase the risk of misinformation, algorithmic bias, and inaccurate clinical reasoning if students lack the skills required to critically evaluate AI outputs [8]. From an educational perspective, this paradox raises important ethical and pedagogical concerns, particularly regarding academic integrity and the potential overreliance on automated technologies in learning environments.

Another important finding concerns differences in AI usage across health disciplines. The study demonstrated significant variation in AI engagement by college affiliation, with dentistry students reporting the longest daily usage durations and the highest practice scores. This finding was further confirmed in the multivariate logistic regression analysis, where both dentistry and medicine students showed significantly higher odds of AI adoption compared with nursing students (Table 8). These differences may reflect discipline-specific technological exposure. Dental education increasingly incorporates digital imaging systems, three-dimensional modeling, and computer-assisted diagnostics, which may encourage students to interact more frequently with AI-related technologies [19]. In contrast, nursing education globally has been slower to integrate AI technologies into curricula, which may explain the comparatively lower practice scores observed among nursing students [9]. These findings suggest that institutional and disciplinary contexts play a critical role in shaping students’ adoption behaviors.

The multivariate regression results also revealed that knowledge score was a significant predictor of AI adoption, whereas gender, academic year, and previous AI training were not significant determinants (Table 8). The positive association between knowledge and adoption aligns with technology acceptance models suggesting that individuals with higher digital literacy are more likely to engage with emerging technologies [20,15]. Conversely, the absence of significant gender differences indicates that AI engagement among health sciences students may be increasingly gender-neutral, reflecting broader trends in digital technology adoption among younger populations. The predominance of female participants in this study likely reflects the demographic structure of health sciences programs, particularly nursing, which traditionally enrolls higher proportions of female students.

Beyond individual determinants, the study identified several structural barriers limiting AI adoption. The most frequently reported obstacles included limited curriculum integration, ethical and privacy concerns, and lack of training centers. These findings are consistent with international evidence showing that institutional readiness, faculty expertise, and technological infrastructure are key determinants of AI integration in health professions education [17,18]. The absence of structured AI training programs within health sciences curricula may explain why students rely heavily on self-directed learning and informal exposure to AI technologies.

Ethical concerns emerged as one of the most prominent barriers reported by students. Nearly half of the participants expressed apprehension regarding privacy risks and ethical implications associated with AI use. These concerns reflect broader debates within digital health and medical education literature regarding data protection, algorithmic bias, transparency of AI systems, and the potential erosion of clinical judgment [13,12]. In the Palestinian context, where digital governance frameworks are still evolving, integrating AI ethics education into health sciences curricula may be essential to ensure responsible and accountable use of emerging technologies.

Contextual factors specific to Palestine may also contribute to the barriers identified in this study. Universities operating in resource-constrained and politically unstable environments often face limitations related to technological infrastructure, internet connectivity, and access to advanced digital tools. Economic constraints may also restrict access to premium AI platforms that require paid subscriptions. These infrastructural challenges may partially explain why students reported limited access to equipment and training opportunities. Similar barriers have been reported in other low-resource educational settings where technological innovation often progresses faster than institutional capacity to implement it effectively [13].

Beyond education, AI technologies are rapidly transforming biomedical research and clinical practice, further emphasizing the importance of AI literacy among health professionals. Machine learning algorithms are increasingly used in medical imaging analysis, cardiovascular diagnostics, and pathology to assist clinicians in identifying patterns within complex datasets [2]. In biomedical research, computational techniques such as bioinformatics analysis and network pharmacology are becoming essential tools for exploring disease mechanisms and identifying therapeutic targets. These developments highlight the growing importance of data science and AI competencies for future healthcare professionals, reinforcing the need for early exposure during undergraduate education.

Overall, the findings of this study suggest that AI adoption among Palestinian health sciences students is characterized by high exposure but limited institutional support. While students appear motivated to use AI technologies in their academic activities, meaningful integration remains constrained by curricular gaps, insufficient training opportunities, and concerns regarding ethical and responsible use. Addressing these barriers will require coordinated institutional strategies, including curriculum redesign, faculty capacity building, investment in digital infrastructure, and development of ethical guidelines for AI use in health professions education.

Collectively, these efforts may help transform AI from an informal academic tool into a structured educational resource that enhances learning, supports clinical decision-making skills, and prepares future healthcare professionals for an increasingly digital healthcare landscape.

### Strengths and limitations

A key strength of this study is its large, diverse sample drawn from three major health sciences colleges, allowing for robust comparisons across disciplines. The use of a validated, multidimensional instrument further enhances the credibility of the findings by capturing multiple components of AI engagement, including usage patterns, barriers, and determinants. Additionally, examining determinants using both Mann–Whitney and Kruskal–Wallis tests provides rigorous statistical insight into demographic and institutional influences.

However, the study is not without limitations. The cross-sectional design restricts causal interpretation, as adoption patterns and barriers may evolve over time with changes in curriculum or technology access. Convenience sampling, although practical in large academic settings, may introduce selection bias by favoring students who are more engaged or technologically active. The reliance on self-reported measures may also lead to response bias, particularly regarding sensitive topics such as ethical concerns or academic use of AI for assignments. Furthermore, the study was conducted at a single institution, which limits generalizability to other Palestinian universities with different infrastructure levels or pedagogical approaches. Finally, although the study explored multiple determinants, qualitative insights were not collected, restricting deeper understanding of students’ motivations or nuanced perceptions.

## Conclusion

This study provides important insights into the barriers, adoption patterns, and determinants of AI use among health sciences students at Palestine Polytechnic University. While AI usage is widespread, students primarily engage with AI for academic and personal purposes rather than professional or clinical tasks. Adoption patterns differ significantly across disciplines, with college affiliation emerging as the strongest determinant of practice scores. Despite the high interest in AI, substantial systemic barriers—most notably limited curriculum integration, ethical concerns, and insufficient training opportunities—continue to impede meaningful and responsible AI adoption.

These findings underscore the urgent need for strategic, institution-wide approaches to AI integration in Palestinian health sciences education. Efforts should prioritize curricular reform, faculty development, ethical guidance, and resource enhancement to ensure that students are equipped with the competencies necessary to navigate an increasingly digital healthcare landscape. Addressing these gaps is essential not only for improving educational quality but also for preparing a digitally competent workforce capable of leveraging AI to enhance patient care, safety, and health system efficiency.
